# Demographics, medication use, and admission characteristics of patients hospitalized with diabetes in Ontario, Canada: A retrospective cohort study

**DOI:** 10.1371/journal.pone.0307581

**Published:** 2024-08-29

**Authors:** Michael Colacci, Afsaneh Raissi, Tor Biering-Sørensen, Michelle Gyenes, Benazir Hodzic-Santor, Saba Manzoor, Kristoffer Skaarup, Jason Moggridge, Ashley Raudanskis, Shohinee Sarma, Fahad Razak, Amol Verma, Michael Fralick

**Affiliations:** 1 Sinai Health System, Division of General Internal Medicine, Toronto, Ontario, Canada; 2 Department of Medicine, University of Toronto, Toronto, Ontario, Canada; 3 St. Michael’s Hospital, University of Toronto, Toronto, Ontario, Canada; 4 Department of Cardiology, Copenhagen University Hospital—Herlev & Gentofte, Copenhagen, Denmark; 5 Institute of Health Policy, Management, and Evaluation, University of Toronto, Toronto, Ontario, Canada; Hamad Medical Corporation, QATAR

## Abstract

**Background:**

In Canada, one in seven adults has diabetes (i.e., 2.3 million) and the lifetime risk of developing diabetes is approximately 30% by age 65. Although 30% of patients admitted to the hospital have diabetes, data from inpatient hospitalizations for patients with diabetes are lacking, both in Canada and globally.

**Objective:**

To validate International Classification of Diseases 10^th^ edition Canadian version (ICD-10-CA) codes for the identification of patients with diabetes, to create a multicenter database of patients with diabetes hospitalized under internal medicine in Ontario, and to determine their baseline characteristics, medication use, and admission characteristics.

**Study design:**

We created a database of people who had diabetes and were hospitalized between 2010 and 2020 at 8 hospitals in Ontario that were part of the General Medicine Inpatient Initiative (GEMINI) hospital data-sharing network. Patients who had diabetes were identified using chart review, based upon either (i) a previous physician diagnosis of diabetes, (ii) a recorded hemoglobin A1c ≥ 6.5% or (iii) outpatient prescription of a diabetes medication preceding the hospitalization. The test characteristics of ICD-10-CA codes for diabetes were evaluated. We compared baseline demographics, medication use and hospitalization details among patients with and without diabetes. For hospitalization details, we collected information on the admission diagnosis, comorbidity index, length of stay, receipt of ICU-level care, and inpatient mortality.

**Results:**

There were 384,588 admissions within the total study cohort, of which 118,987 (30.9%) had an ICD-10-CA diagnosis code of diabetes (E10.x, E11.x, E13.x, E14.x). The sensitivity and specificity of ICD-10-CA diagnostic codes was 95.9% and 98.8%, respectively. Most patients with an ICD-10-CA code for diabetes had a code for type 2 diabetes (93.9%) and a code for type 1 diabetes was rare (6.1%). The mean age was 66.4 years for patients without diabetes and 71.3 years for those with an ICD-10-CA diagnosis code for diabetes. Patients with diabetes had a higher prevalence of hypertension (64% vs. 37.9%), coronary artery disease (28.7% vs. 15.3%), heart failure (24.5% vs. 12.1%) and renal failure (33.8% vs. 17.3%) in comparison to those without diabetes. The most prevalent diabetes medications received in hospital were metformin (43%), DPP4 inhibitors (22.7%) and sulfonylureas (18.8%). The most common reason for admission among patients with diabetes was heart failure (9.0%), and among patients without diabetes was pneumonia (7.8%). Median length of stay was longer for patients with diabetes (5.5 vs. 4.5 days) and in-hospital mortality was similar between groups (6.8% with diabetes vs. 6.5% without diabetes).

**Importance:**

Diabetes is one of the most prevalent chronic medical conditions, affecting roughly one third of all patients hospitalized on an internal medicine ward and is associated with other comorbidities and longer hospital stays. ICD-10-CA codes were highly accurate in identifying patients with diabetes. The development of an inpatient cohort will allow for further study of in-hospital practices and outcomes among patients with diabetes.

## Introduction

Over 400 million people around the world have diabetes mellitus [1]. In Canada, one in seven adults has diabetes (i.e., 2.3 million) and the risk of developing diabetes by age 65 is roughly 30% [1]. Patients with diabetes have an increased risk of hospitalization [2]. Despite this, most large administrative databases (e.g., Institute of Clinical Evaluative Sciences (ICES), Denmark, Sweden, US insurance claims) do not capture most inpatient data [3, 4]. One reason this problem exists is because hospitals use different electronic medical record systems, and thus extracting data from the inpatient setting cannot be easily automated.

The limited data from the inpatient setting means little is known about what occurs when people with diabetes are hospitalized, and without this data it is challenging to develop strategies to improve inpatient care. This is reflected in international guidelines (e.g., National Institute for Health and Care Excellence [NICE], Diabetes Canada, American Diabetes Association) that often cite low-quality evidence for inpatient management strategies [5–7]. For example, it is well established that sodium-glucose cotransporter 2 (SGLT2) inhibitors and glucagon-like peptide 1 (GLP1) receptor agonists reduce the risk of cardiovascular events and potentially all-cause mortality, but these medications are seldom started prior to discharge from hospital [8]. As another example, severe hypoglycemia can be life-threatening, but mechanisms to identify patients at highest risk during their hospitalization are lacking [9]. The absence of inpatient data means that many of the decisions we make are not supported by high quality evidence, and until inpatient data are readily available and accessible, both basic and more complex inpatient clinical questions cannot be answered. This is particularly important given that over 30% of patients admitted to the general internal medicine ward have a history of diabetes [10].

The objective of our study was to evaluate the accuracy of inpatient International Classification of Diseases 10^th^ edition Canadian version (ICD-10-CA) codes for the diagnosis of diabetes, to create a multicenter database of patients with diabetes hospitalized under internal medicine in Ontario, and to determine their baseline characteristics, medication use, and admission characteristics.

## Methods

### Study design

We conducted a cohort study of adults admitted to the inpatient general internal medicine service between January 1, 2010 and June 1, 2020 at 8 hospitals in Ontario, Canada that are part of the General Medicine Inpatient Initiative (GEMINI) database [10]. The GEMINI database includes administrative data (at both the patient and provider level) and clinical data for all patients admitted to, or discharged from, the general internal medicine units at the affiliated sites [10].

### Cohort definition and follow up

We included all consecutive adult patients (≥ 18 years old) admitted to or discharged from the inpatient general internal medical service or medical-surgical intensive care unit (ICU) between January 1, 2010 and June 1, 2020. The general internal medicine service accounts for up to 40% of emergency admissions to hospital and nearly 25% of hospital bed-days. It is the largest single group of hospitalized patients and includes most patients with medical indications for hospitalization [10]. Patients were followed from the date of hospital admission to the first of either hospital discharge or death.

### Data sources

Administrative and clinical data were extracted from each patient’s electronic medical record through methods previously described by the GEMINI research program [11]. Patients’ electronic medical records were linked to the following administrative datasets: the Canadian Institute for Health Information (CIHI) Discharge Abstract Database (DAD) and the National Ambulatory Care Reporting System (NACRS). Linkage through these databases provides information on their past medical history pre-dating their hospitalization as well as administrative data for their current hospitalization. Specifically, CIHI and NACRS provides ICD-10-CA codes which can be utilized to understand both their past medical history and the diagnoses for their current hospitalization. All data were accessed through the GEMINI database between November 30, 2020, and May 15, 2023.

### ICD-10-CA code abstraction

During each hospital admission, diagnoses are submitted to the DAD within CIHI, including the most responsible diagnosis (MRDx), comorbidity diagnoses, secondary diagnoses, admitting diagnoses and service transfer diagnosis [12, 13]. Each diagnosis is encoded with an ICD-10-CA code [14]. The MRDx is the one diagnosis that is most responsible for the patient’s stay in hospital; if there is more than one condition, it is the diagnosis that contributed to the greatest proportion of the length of stay [13]. Comorbidity diagnoses are separated into pre-admit diagnoses (existing prior to hospitalization), and post-admit diagnoses (arose after admission and during hospitalization) [13]. Secondary diagnoses are diagnoses or conditions for which the patient may or may not have received treatment and do not meet any of the criteria for significance (i.e., require treatment beyond maintenance of the pre-existing condition, increase the length of stay by at least 24 hours, or significantly affect the treatment received) [13]. The admitting diagnosis is an optional diagnosis or condition that can be assigned if the reason for admission differs from the most responsible diagnosis [13]. Finally, the service transfer diagnosis is the diagnostic code associated with a patient transfer between services, or between levels of care (e.g., transfer to alternate level of care [ALC]) [13].

### Clinical data abstraction

Other details obtained from the current hospitalization included demographic information (age, sex, proxies for socioeconomic status [e.g., neighbourhood income quintile, place of residence prior to hospitalization]), length of stay, receipt of ICU level care, and inpatient mortality. Clinical data from the hospitalization was available within GEMINI and directly extracted from the patient’s electronic medical record through a data transfer. Clinical data included their past medical history (using Clinical Classification Software Refined [CCSR] codes, e.g., hypertension, coronary artery disease), diabetes severity (e.g., hemoglobin A1c), diabetes complications (e.g., nephropathy), inpatient medications (e.g., insulin, metformin), laboratory values (e.g., creatinine), imaging results, vital signs, and physician orders. The accuracy of these data relative to manual chart review by a trained chart abstractor have previously been evaluated and all data fields have an accuracy of above 99% [11]. The S1 Appendix provides further details on the criteria used to identify these prior diagnoses. Medications received and laboratory values obtained prior to the time of presentation to the hospital (which mainly occurred through the emergency department) were not available within GEMINI and thus not included in the overall analysis, but manually chart-abstracted for a subset of patients.

### Manual chart review

In addition to the administrative and clinical data automatically captured from the GEMINI database, charts were also manually reviewed by trained abstractors to obtain other datapoints that were not available within GEMINI. We performed a manual chart review of 650 randomly selected charts, including 500 that were reviewed for the presence or absence of an ICD-10-CA code for diabetes and 650 that were evaluated for a glucose ≥ 11.1 mmol/L. The admission note, discharge summary, lab values and pharmacy notes were manually reviewed to determine whether the patient had a diagnosis of diabetes. A patient was considered to have diabetes if they met any one of the following criteria: (1) a physician diagnosis of diabetes in their admission note or discharge note, (2) prescription of a home diabetes medication (S1 Appendix) in their admission or pharmacy note, or (3) a recorded hemoglobin A1c value of 6.5% or greater in the admission note or laboratory values. This served as the ground truth definition of diabetes within our study to which other criteria were compared. Additional data not available within GEMINI and collected through manual chart review included body mass index (BMI), smoking status, pre-hospitalization medications, and discharge medications.

### Study objectives

The primary objective of our study was to evaluate the accuracy of inpatient ICD-10-CA codes for the diagnosis of diabetes with the goal of creating a multicentre database of patients with diabetes who were hospitalized in Ontario. Secondary objectives included describing the baseline characteristics, medication use, and reason for hospitalization among adults with diabetes. Secondary outcomes included the length of hospital stay, ICU utilization and inpatient mortality.

### Statistical analysis

We calculated descriptive statistics for demographic variables (e.g., age, sex, income quintile), comorbidities, inpatient medications and baseline laboratory values. We calculated standardized differences to compare the frequency of comorbidities (e.g., coronary artery disease, congestive heart failure and cerebrovascular disease), medication use (metformin, a dipeptidyl-peptidase 4 [DPP4] inhibitors and SGLT2 inhibitors), most responsible discharge diagnosis and laboratory values, between patients with and without diabetes. A standardized difference of 0.1 or less indicates balance between groups [15]. We calculated test characteristics using the following formulae: sensitivity = (true positives/[true positives + false negatives]), specificity = (true negatives/[true negatives + false positives]), positive predictive value = (true positives/[true positive + false positives]), and negative predictive value = (true negatives/[true negatives + false negatives]). The test criteria was an ICD-10-CA code, which was compared to the ground truth criteria of a physician diagnosis of diabetes, receipt of a diabetes medication prior to hospitalization or hemoglobin A1c ≥ 6.5%, as detailed above. R version 4.1.3 was utilized for all analyses [16].

### Ethics

Research Ethics Board approval was obtained from St. Michael’s Hospital on behalf of all participating hospitals, with a waiver of patient consent for this retrospective study using routinely collected health data.

### Funding

The funders had no role in study design, data collection and analysis, decision to publish, or preparation of the manuscript.

## Results

We identified 384,588 patient admissions to one of 8 hospitals in Ontario between the years of 2010 and 2020 (Table 1). In comparison to manual chart review ICD-10-CA codes for diabetes typically had sensitivity and specificity greater than 90%, though this varied depending on the position of the ICD-10-CA code (Table 2). When an ICD-10-CA code for diabetes in only the MRDx position was utilized, the sensitivity was 80.3% and the specificity was 100%. In contrast, when an ICD-10-CA code at any diagnostic position was used, the specificity remained high at 98.8% and the sensitivity increased to 95.9%. As noted, a person was considered to have diabetes on manual chart review if their chart contained either a physician diagnosis of diabetes, a hemoglobin A1c value equal to or greater than 6.5%, or a prescription for a diabetes medication prior to hospitalization. Hemoglobin A1c measurements were completed during the hospitalization in 12.9% of all patients within the cohort, and 21.3% of patients with an ICD-10-CA code for diabetes. Among patients with diabetes, 75.5% received any diabetes medication while in hospital. Based on the sensitivity and specificity noted above, data missingness and feasibility of deployment, the definition of diabetes utilized to create the inpatient cohort was an ICD-10-CA code for diabetes mellitus in any diagnostic position (S1 Appendix).

**Table 1:** Characteristics of patients admitted to the internal medicine ward with and without a prior diagnosis of diabetes.

	No Diabetes (N = 265,601)	Diabetes (N = 118,987)	SD
**Age** (years), mean (IQR)	66.4 (53–83)	71.3 (63–82)	0.28
<40 N(%)	33,786 (12.7%)	4,114 (3.46%)	
40–50	22,096 (8.32%)	5,578 (4.69%)	
50–60	34,822 (13.1%)	13,710 (11.5%)	
60–70	41,427 (15.6%)	23,956 (20.1%)	
70–80	46,052 (17.3%)	31,642 (26.6%)	
80–90	59,279 (22.3%)	31,019 (26.1%)	
90–100	27,131 (10.2%)	8,778 (7.38%)	
≥100	1,008 (0.38%)	190 (0.16%)	
**Female,** N(%)	134,329 (50.6%)	53,844 (45.3%)	0.107
**Neighbourhood Income quintile,** N(%)			0.168
One (lowest)	50,403 (19%)	27,522 (23.1%)	
Two	45,971 (17.3%)	22,483 (18.9%)	
Three	45,770 (17.2%)	22,086 (18.6%)	
Four	48,205 (18.1%)	20,879 (17.5%)	
Five (highest)	57,363 (21.6%)	19,330 (16.2%)	
Missing	17,889 (6.7%)	6,687 (5.6%)	
**LTC resident,** N(%)	8,553 (3.2%)	5,741 (4.8%)	0.082
**Chronic disease,** N(%)			
Hypertension	100,641 (37.9%)	76,142 (64%)	0.541
Coronary artery disease	40,586 (15.3%)	34,118 (28.7%)	0.328
Peripheral vascular disease	7,518 (2.8%)	3,780 (3.2%)	0.02
Retinopathy	4,618 (1.7%)	4,103 (3.5%)	0.108
Neuropathy	2,804 (1.1%)	7,173 (6.0%)	0.271
Diabetic foot infection	5,758 (2.2%)	59,434 (49.9%)	1.298
Obesity	2,807 (1.1%)	4,507 (3.8%)	0.178
Hyperlipidemia	30,633 (11.5%)	30,453 (25.6%)	0.368
COPD/Asthma	37,654 (14.2%)	19,492 (16.4%)	0.061
Dementia	48,641 (18.3%)	28,805 (24.2%)	0.144
Liver failure	12,332 (4.6%)	7,814 (6.6%)	0.084
Renal failure	45,947 (17.3%)	40,250 (33.8%)	0.386
Stroke or TIA	15,983 (6.0%)	11,219 (9.43%)	0.128
Heart failure	32,170 (12.1%)	29,104 (24.5%)	0.324
**Diabetes specific**			
Type 1 diabetes, N(%)	-	7,264 (6.1%)	0.359
Type 2 diabetes, N(%)	-	111,906 (93.9%)	4.967
Hemoglobin A1C (%), median (IQR)	5.6 (5.3–6) [90.9%]	7.3 (6.3–8.9) [78.7%]	1.328
Highest glucose in hospital (mmol/L), median (IQR)	6.7 (5.7–8.2) [4.6%]	12.1 (8.6–17.4) [2.1%]	1.122
Lowest glucose in hospital (mmol/L), median (IQR)	5.4 (4.8–6.3) [4.6%]	5.9 (4.6–8) [2.1%]	0.39
**Inpatient Medication Use,** N(%)			
Metformin	2,028 (0.8%)	51,147 (43%)	1.188
DPP4	652 (0.3%)	27,013 (22.7%)	0.753
Sulfonylurea	2,433 (0.9%)	22,325 (18.8%)	0.628
Insulin	6,775 (2.6%)	21,494 (18.1%)	0.528
GLP1	16 (0.006%)	132 (0.1%)	0.043
SGLT2	103 (0.04%)	2,229 (1.9%)	0.189
**Baseline laboratory values,** median (IQR)			
Sodium (mmol/L)	138 (134–140) [1.9%]	137 (133–139) [1%]	0.154
Glucose (mmol/L)	6.2 (5.4–7.3) [4.6%]	9.1 (6.8–13) [2.1%]	0.868
Hemoglobin (g/L)	125 (108–140) [1.8%]	119 (103–135) [1%]	0.197
Potassium (mmol/L)	4 (3.7–4.4) [2%]	4.3 (3.9–4.7) [1%]	0.334
Lactate (mmol/L)	1.6 (1.1–2.4) [50.8%]	1.9 (1.3–2.9) [43.9%]	0.153
LDL (mmol/L)	2.1 (1.5–2.9) [94.6%]	1.6 (1.1–2.4) [93.8%]	0.38
Leukocytes (x10^9^/L)	8.9 (6.6–12.4) [1.9%]	9.4 (7.1–12.8) [1%]	0.053
Platelets (x10^9^/L)	225 (170–292) [1.9%]	226 (172–293) [1.1%]	0.008
Total cholesterol (mmol/L)	3.8 (3.0–4.7) [90.1%]	3.3 (2.7–4.2) [88.1%]	0.297
CRP (mg/L)	29 (6–94) [89.2%]	41 (9–113) [90.1%]	0.139
Creatinine (μmol/L)	81 (65–110) [2%]	106 (76–165) [1.1%]	0.358
ESR (mm/h)	33 (15–59) [90.2%]	49 (25–81) [90.9%]	0.404

SD: Standardized Difference, IQR: Interquartile range, N: Number, LTC: Long term care, COPD: Chronic obstructive pulmonary disease, TIA: Transient ischemic attack, DPP4: Dipeptidyl-peptidase 4 inhibitor, GLP1: Glucagon-like peptide 1 receptor agonist, SGLT2: Sodium-glucose cotransporter 2 inhibitor, LDL: Low-density lipoprotein, CRP: C-reactive protein, ESR: Erythrocyte sedimentation rate. Percentages in square brackets [%] indicate proportion of missing values.

**Table 2:** Test characteristics of ICD-10-CA codes using CIHI categories for diagnosis of diabetes mellitus.

Criteria	Sensitivity	Specificity	Positive Predictive Value	Negative Predictive Value
Diabetes ICD-10-CA code as MRDx	80.3%	100%	100%	85.3%
Diabetes ICD-10-CA code in any position	95.9%	98.8%	98.6%	96.5%
Glucose > 11.1 (mmol/L)	97.3%	34.5%	69.3%	89.5%

ICD-10-CA: International Classification of Diseases 10^th^ edition Canadian version, CIHI: Canadian Institute for Health Information, MRDx: Most responsible diagnosis. Test characteristics were calculated using the test criteria listed in column 1 compared to the gold standard definition for diabetes, which was patient chart review with any of (1) a physician diagnosis of diabetes in their admission or discharge note, (2) prescription of a diabetes medication in their admission or pharmacy note, or (3) a recorded hemoglobin A1c value greater than or equal to 6.5% in the admission note or laboratory values. ICD-10-CA codes utilized were E10.x for Type 1 Diabetes and E11.x, E13.x and E14.x for Type 2 Diabetes.

### ICD-10-CA diabetes cohort

#### Patient characteristics

Using the presence of an ICD-10-CA code to define diabetes, the mean age was 71.3 years for patients with diabetes and 66.4 years for patients without diabetes (Table 1). Sex distribution was similar between the two groups (45.3% female with diabetes vs. 50.6% female without diabetes, Standardized Difference [SD] = 0.1) as was residence in a long-term care home (4.8% with diabetes vs. 3.2% without diabetes, SD = 0.08). Patients with diabetes were more likely to have heart failure (24.5% vs. 12.1%, SD = 0.32) and dementia (24.2% vs. 18.3%, SD = 0.14), but not chronic obstructive pulmonary disease/asthma (16.4% vs. 14.2%, SD = 0.06). Patients with diabetes had a higher median hemoglobin A1c (7.3% Interquartile Range [IQR] 6.3–8.9%, vs. 5.6% IQR 5.3–6.0%, SD = 1.33) and creatinine value (106 IQR 76–165 μmol/L vs. 81 IQR 65–110 μmol/L, SD = 0.36).

#### Medication use

During their hospitalization, 43% of patients with diabetes received metformin, 22.7% received a DPP4 inhibitor, 18.8% received a sulfonylurea, 18.1% received insulin, 1.9% received an SGLT2 inhibitor and 0.1% received a GLP1 receptor agonist (Table 1). Empagliflozin was the most common SGLT2 inhibitor, accounting for 59.3% of all SGLT2 inhibitors prescribed.

#### Admission characteristics

Among patients hospitalized with a prior diagnosis of diabetes, heart failure was the most common admission diagnosis (9.0%), followed by pneumonia (7.8%) and complication of diabetes (7.5%) (Table 3). Among patients without a prior diagnosis of diabetes, the most common reason for admission was pneumonia (7.8%), followed by bacterial infections (6.2%), and urinary tract infections (5.1%).

**Table 3:** Characteristics of hospital admissions and outcomes among patients admitted with and without a history of diabetes mellitus.

	Patients with diabetes	Patients without diabetes
Most Responsible Diagnosis, N(%)		
1	Heart failure 10748 (9.0%)	Pneumonia 20960 (7.8%)
2	Pneumonia 9319 (7.8%)	Bacterial infection 16577 (6.2%)
3	Diabetes complication 8917 (7.5%)	UTI 13411 (5.1%)
4	Bacterial infection 8733 (7.3%)	Heart failure 12852 (4.8%)
5	UTI 7422 (6.2%)	COPD/Asthma 12281 (4.6%)
Length of Stay (days), median (IQR)	5.5 (2.7–10.7)	4.5 (2.1–9.2)
Intensive care unit utilization, N(%)	15,538 (13.1%)	30,855 (11.6%)
Death in hospital, N(%)	8,122 (6.83%)	17,280 (6.51%)

UTI: Urinary Tract Infection, IQR: Interquartile range, COPD: Chronic obstructive pulmonary disease.

#### Outcomes

The median length of stay was 5.5 (Interquartile Range [IQR] 2.7–10.7) days for patients with diabetes in comparison to 4.5 (IQR 2.1–9.2) days for patients without diabetes (Table 3). The proportion of patients with diabetes admitted to the ICU during their hospitalization was 13.1% in comparison to 11.6% among patients without diabetes. Inpatient mortality was 6.8% for patients with diabetes versus 6.5% among patients without diabetes.

### Data from manual chart abstraction

Within the subset of diabetes patients for which manual chart abstraction was performed, rates of medication use prior to hospitalization were as follows: insulin 50%, metformin 42%, DPP4 inhibitor 27%, sulfonylurea 14%, SGLT2 inhibitor 5.1% and GLP1 receptor agonist <1% (Table 4). Rates of medication prescription at discharge were as follows: insulin 57%, metformin 44%, DPP4 inhibitor 28%, sulfonylurea 12%, SGLT2 inhibitor 3.5%, and GLP1 receptor agonist <1%. Sulfonylureas and SGLT2 inhibitors were the two medications most frequently discontinued during hospitalization. Among all patients taking a sulfonylurea prior to hospitalization, 19% had the sulfonylurea discontinued and not restarted at the time of discharge. Among all patients taking an SGLT2 inhibitor prior to hospitalization, 32% had the SGLT2 inhibitor discontinued and not restarted at the time of discharge. Within the abstracted chart dataset, it was identified that 6.5% of patients with diabetes were diagnosed with diabetes during the hospitalization (S1 Appendix). BMI among patients with a weight and height measurement during the hospitalization was 29.4 kg/m2 among patients with diabetes and 26.0 kg/m2 for patients without diabetes (S1 Appendix).

**Table 4:** Diabetes medications prescribed prior to hospitalization and at the time of discharge according to manual chart review.

	Diabetes (N = 370)	No diabetes (N = 280)
**Home Medication** ^a^	N (%)	N (%)
Metformin	155 (42%)	<5 (<1%)
Insulin	185 (50%)	<5 (<1%)
DPP4	99 (27%)	0%
Sulfonylurea	53 (14%)	0%
SGLT2	19 (5.1%)	0%
GLP-1	<5 (<1%)	0%
**Discharge Medication** ^b^		
Metformin	164 (44%)	<5 (<1%)
Insulin	211 (57%)	<5 (<1%)
DPP4	104 (28%)	0%
Sulfonylurea	43 (12%)	0%
SGLT2	13 (3.5%)	0%
GLP-1	<5 (<1%)	0%

^a^: Home medication denotes patient was prescribed the medication prior to hospitalization.

^b^: Discharge medication indicates the patient was recommended to continue taking this medication after discharge from the hospital. DPP4: Dipeptidyl-peptidase 4 inhibitor, GLP-1: Glucagon-like peptide 1 receptor agonist, SGLT2: Sodium-glucose cotransporter 2 inhibitor. Cells with <5 observations were labelled as such to protect potentially identifiable patient information.

## Discussion

In this study of 8 hospitals and over 380,000 total hospital admissions, there were 118,987 admissions among patients with diabetes. ICD-10-CA codes for diabetes had a sensitivity and specificity greater than 90% measured against manual chart review. Patients with diabetes accounted for 30.9% of all hospital admissions to internal medicine. The most frequent reason for admission among patients with diabetes was heart failure, followed by pneumonia. The average length of stay among patients with diabetes was 5.5 days, and 6.8% died in hospital. This large multicentre inpatient diabetes database will allow for the evaluation of multiple components of diabetes management in hospital.

Patients with diabetes in this cohort were commonly admitted for conditions related to cardiovascular, renal and infectious diseases. These diseases are likely to be both directly and indirectly related to their diabetes. This aligns with prior literature showing that admissions for skin and soft tissue infection, urinary tract infection, stroke, and electrolyte abnormalities were higher in patients with diabetes compared to those without diabetes [17]. Two new classes of medications (SGLT2 inhibitors and GLP1 receptor agonists) reduce the risk of cardiovascular and renal events in patients with diabetes [8, 18]. Use of these medications was exceedingly rare in our cohort which we suspect is due to several reasons. First, many of the admissions in this study occurred prior to the availability of these medications in Ontario. Second, the uptake of these medications into clinical practice has been slow, especially in the inpatient setting. For example, in a large US population-based study, only a small portion of eligible patients had been started on these newer medications [19]. Ensuring that patients are on optimal guideline-directed pharmacologic therapy may be an important focus for physicians and hospital groups to improve patient care, symptom burden, and frequency of hospitalization. This inpatient diabetes database will help to better understand safety and adverse effects of new pharmacotherapies in real world practice.

Prior studies have utilized diagnostic codes, and in particular, ICD-9 or ICD-10 codes, to identify patients with diabetes [20, 21]. We found that ICD-10-CA codes for diabetes as the most responsible diagnosis had a specificity exceeding 99%, but a sensitivity of 80%. In contrast, ICD-10-CA diagnosis codes for diabetes at any position (i.e., not just the most responsible diagnosis code) had a sensitivity of 96% and specificity of 99% for the diagnosis of diabetes, measured against the gold-standard of manual chart review. Our results suggest that evaluating for an ICD-10-CA code for diabetes at any diagnosis position for an inpatient admission (most responsible diagnosis, comorbidity diagnosis, secondary diagnosis and admitting diagnosis) is a highly sensitive and specific method for identifying patients with diabetes in health administrative databases.

People with diabetes are three times more likely to be hospitalized compared to age-matched adults who do not have diabetes [17]. By creating a centralized database to house hospitalization data, GEMINI-DM will allow us to gain insight and understanding into the quality of the care provided in inpatient centers as well as clinical outcomes following hospitalization for patients with diabetes. As we gather more information about these inpatient hospitalizations, we can identify gaps and improve the care of this patient population. To measure pre- and post-hospital events, GEMINI-DM will be linked to ICES, which contains longitudinal health-related data (e.g., anonymous patient records, population-based health surveys, and clinical data) for all residents of Ontario. Thus, the GEMINI-DM cohort linked with ICES will allow us to understand how treatment decisions can affect a person’s risk of hospitalization and how a hospitalization can impact what happens after a patient is discharged home. These rich data will be able to answer a wide range of research questions including, but not limited to, quality of inpatient care of people with diabetes, clinical effectiveness of treatment strategies implemented during a hospitalization, and evaluation of clinical prediction tools to prevent adverse events during an inpatient hospitalization.

Our study has several limitations. First, our cohort primarily included adults hospitalized under the internal medicine service, and therefore may not reflect characteristics of inpatients with diabetes admitted to pediatric, surgical or psychiatric units. Second, there are several rare conditions (e.g., iron deficiency, hemolysis) which can falsely elevate or lower the hemoglobin A1c value. However, because these conditions are relatively rare, we do not believe this would significantly affect the classification of patients as with or without diabetes according to hemoglobin A1c value. Third, the reason for prescribing a medication was not available for all patients. Some patients may have received metformin, an SGLT2 inhibitor or GLP1 receptor agonist for reasons other than diabetes (such as prediabetes, PCOS, heart failure and obesity, respectively). However, during the study time period, neither SGLT2 inhibitors nor GLP1 receptor agonists were approved for patients without diabetes, and this is therefore likely to be rare. Similarly, given the age distribution of patients in our study, use of metformin for PCOS is expected to be low. Fourth, identification of whether patients were diagnosed with diabetes during or prior to the hospitalization was based on chart review and only available for a subsample of the entire cohort. We therefore could not stratify results by whether patients had a new or existing diagnosis of diabetes. Finally, our cohort primarily included large hospitals in urban settings and thus our results may not generalize to rural hospitals.

## Conclusion

In summary, patients with diabetes represent 30% of all admissions to internal medicine, have a longer length of stay, and have high rates of ICU admission and inpatient mortality. Cardiovascular, infectious, and renal complications of diabetes are the most frequent reasons for admission, and many patients are not taking therapies shown to decrease the risk of admission for these complications. Future research should focus on preventing and managing complications among patients with diabetes and increasing utilization of the most effective pharmacologic and non-pharmacologic interventions.

## Supporting information

S1 Checklist Checklist of PLOS one required manuscript items. (DOCX)

S1 Appendix Appendix with supplementary methods, tables and figures referenced in the manuscript, as well as the STROBE observational study checklist. (DOCX)
