# Supplementary material for: Demographics, medication use, and admission characteristics of patients hospitalized with diabetes in Ontario, Canada: A retrospective cohort study
Source: PLoS One. 2024 Aug 29;19(8):e0307581. doi: 10.1371/journal.pone.0307581 (PMC11361565; doi:10.1371/journal.pone.0307581)
Supplement: S1 Appendix — (DOCX) [file pone.0307581.s002.docx]

**APPENDIX**

Supplemental Table 1: Data Extraction source for all Table 1 variables

|  | **VARIABLE SOURCE** | **CCSR codes** |
| --- | --- | --- |
| **Age** |  |  |
| Year, mean+/- SD, range | GEMINI | NA |
| < 40 | GEMINI | NA |
| 40 – 60 | GEMINI | NA |
| 61 – 80 | GEMINI | NA |
| 81 – 100 | GEMINI | NA |
| > 100 | GEMINI | NA |
| **Sex, Female** | GEMINI | NA |
| **Neighbourhood Income quintile** |  |  |
| One (lowest) | GEMINI | NA |
| Two | GEMINI | NA |
| Three | GEMINI | NA |
| Four | GEMINI | NA |
| Five (highest) | GEMINI | NA |
| Missing | GEMINI | NA |
| **LTC resident** | GEMINI | NA |
| **Chronic disease** |  |  |
| Type 1 diabetes | GEMINI | END004 |
| Type 2 diabetes | GEMINI | END002, END003, END005 |
| Hypertension | GEMINI | CIR007, CIR008 |
| Coronary artery disease | GEMINI | CIR009, CIR010, CIR011 |
| Peripheral vascular disease | GEMINI | CIR026, INJ014, INJ051, INJ052 |
| Retinopathy | GEMINI | EYE005, EYE010 |
| Neuropathy | GEMINI | NVS015 |
| Diabetic foot infection | GEMINI | CIR028, MUS002 |
| Obesity | GEMINI | END009 |
| Hyperlipidemia | GEMINI | END010 |
| COPD/Asthma | GEMINI | RSP008, RSP009 |
| Liver failure | GEMINI | DIG018, DIG019 |
| Renal failure | GEMINI | GEN002, GEN003, GEN010 |
| Stroke or TIA | GEMINI | CIR020, CIR021, CIR022, CIR023, CIR024, CIR025, NVS012 |
| Heart failure | GEMINI | CIR019 |
| Dementia | GEMINI | NVS011 |
| **Diabetes specific** |  |  |
| Hemoglobin A1C | GEMINI | NA |
| Highest glucose in hospital | GEMINI | NA |
| Lowest glucose in hospital | GEMINI | NA |
| Metformin use in hospital | GEMINI | NA |
| DPP4 use in hospital | GEMINI | NA |
| Sulfonylurea use in hospital | GEMINI | NA |
| Insulin use in hospital | GEMINI | NA |
| GLP1 use in hospital | GEMINI | NA |
| SGLT2i use in hospital | GEMINI | NA |
| **Most responsible discharge diagnosis** |  |  |
| Heart failure | GEMINI | NA |
| Pneumonia | GEMINI | NA |
| Bacterial infection | GEMINI | NA |
| COPD | GEMINI | NA |
| UTI | GEMINI | NA |
| Diabetes complication | GEMINI | NA |
| **Baseline labs (median, IQR)** |  |  |
| Sodium (mmol/L) | GEMINI | NA |
| Glucose (mmol/L) | GEMINI | NA |
| Hemoglobin (g/L) | GEMINI | NA |
| Potassium (mmol/L) | GEMINI | NA |
| Lactate (mmol/L) | GEMINI | NA |
| LDL (mmol/L) | GEMINI | NA |
| Leukocytes (x10^9^/L) | GEMINI | NA |
| Platelets (x10^9^/L) | GEMINI | NA |
| Total cholesterol (mmol/L) | GEMINI | NA |
| CRP (mg/L) | GEMINI | NA |
| Creatinine (µmol/L) | GEMINI | NA |
| ESR (mm/h) | GEMINI | NA |

Legend: CCSR: Clinical Classification Software Refined, SD: Standardized Difference, IQR: Interquartile range, N: Number, LTC: Long term care, COPD: Chronic obstructive pulmonary disease, TIA: Transient ischemic attack, UTI: Urinary tract infection, DPP4: Dipeptidyl-peptidase 4 inhibitor, GLP1: Glucagon-like peptide 1 receptor agonist, SGLT2: Sodium-glucose cotransporter 2 inhibitor, LDL: Low-density lipoprotein, CRP: C-reactive protein, ESR: Erythrocyte sedimentation rate.

Supplemental Table 2: List of diabetes medications used to determine whether a patient was prescribed a diabetes medication prior to hospitalization

| **DM Drug Category** | **Individual Medications** |
| --- | --- |
| Biguanide | metformin |
| SGLT-2 | canagliflozin, dapagliflozin, empagliflozin, ertugliflozin |
| SGLT-2 combination pills | canagliflozin and metformin, dapagliflozin and metformin, empagliflozin and linagliptin, empagliflozin and metformin hydrochloride, dapagliflozin and saxagliptin hydrochloride, dapagliflozin and metformin hydrochloride and saxagliptin hydrochloride, empagliflozin and linagliptin and metformin hydrochloride, ertugliflozin and metformin hydrochloride, ertugliflozin and sitagliptin phosphate |
| DPP-4 | alogliptin, linagliptin, saxagliptin, sitagliptin, Vildagliptin |
| DPP-4 combination pills | alogliptin and metformin, alogliptin and pioglitazone, empagliflozin and linagliptin, linagliptin and metformin hydrochloride, empagliflozin and linagliptin and metformin hydrochloride, dapagliflozin and saxagliptin hydrochloride, dapagliflozin and metformin hydrochloride and saxagliptin hydrochloride, saxagliptin and metformin, sitagliptin and metformin, sitagliptin and simvastatin, ertugliflozin and sitagliptin phosphate |
| GLP-1 | albiglutide, dulaglutide, exenatide, liraglutide, lixisenatide, Semaglutide |
| GLP-1 combination pills | insulin degludec and liraglutide, insulin glargine and lixisenatide |
| Sulfonylureas | chlorpropamide, glimepiride, glipizide, glyburide, glyburide micronized, tolazamide, tolbutamide, acetohexamide, gliquidone, gliclazide |
| Sulfonylureas combination pills | glipizide and metformin, glyburide and metformin, pioglitazone and glimepiride, glimepiride and rosiglitazone maleate |
| thiazolidinediones | rosiglitazone, pioglitazone, troglitazone |
| thiazolidinediones combination pills | metformin hydrochloride and pioglitazone hydrochloride, glimepiride and pioglitazone hydrochloride, alogliptin benzoate and pioglitazone hydrochloride, metformin hydrochloride and rosiglitazone maleate, glimepiride and rosiglitazone maleate |

Legend: DPP4: Dipeptidyl-peptidase 4 inhibitor, GLP1: Glucagon-like peptide 1 receptor agonist, SGLT2: Sodium-glucose cotransporter 2 inhibitor

Supplemental Table 3. Manual chart review data of new diabetes diagnosis, BMI, obesity and tobacco use.

| **Variable** | **Diabetes (N=370)** | **No diabetes (N=280)** |
| --- | --- | --- |
| New diagnoses of diabetes during hospitalization | 24 (6.5%) | 0 (0%) |
| BMI (kg/m^2^), mean(IQR) | 29.4 (25.2-33.2) [83% N/A] | 26 (22-28.6) [86% N/A] |
| Obesity,(%) | 40 (10.8%) | 12 (4.3%) |
| Current or Previous Tobacco Use, (%) | 30 (8.1%) | 43 (15.4%) |

Legend: BMI: Body Mass Index, IQR: Interquartile range. Percentages in square brackets [%] indicate proportion of missing values where BMI (weight and height), obesity diagnosis or tobacco use were not noted in the medical record.

STROBE Statement—GEMINI-DM, Michael Colacci, May 15, 2023

|  | Item No. | Recommendation | Page  No. | Relevant text from manuscript |
| --- | --- | --- | --- | --- |
| **Title and abstract** | 1 | (*a*) Indicate the study’s design with a commonly used term in the title or the abstract | 1 |  |
|  |  | (*b*) Provide in the abstract an informative and balanced summary of what was done and what was found | 1 |  |
| Introduction | | | |  |
| Background/rationale | 2 | Explain the scientific background and rationale for the investigation being reported | 2 |  |
| Objectives | 3 | State specific objectives, including any prespecified hypotheses | 3 |  |
| Methods | | | |  |
| Study design | 4 | Present key elements of study design early in the paper | 3 |  |
| Setting | 5 | Describe the setting, locations, and relevant dates, including periods of recruitment, exposure, follow-up, and data collection | 3 |  |
| Participants | 6 | (*a*) *Cohort study*—Give the eligibility criteria, and the sources and methods of selection of participants. Describe methods of follow-up  *Case-control study*—Give the eligibility criteria, and the sources and methods of case ascertainment and control selection. Give the rationale for the choice of cases and controls  *Cross-sectional study*—Give the eligibility criteria, and the sources and methods of selection of participants | 3 |  |
|  |  | (*b*) *Cohort study*—For matched studies, give matching criteria and number of exposed and unexposed  *Case-control study*—For matched studies, give matching criteria and the number of controls per case | N/A |  |
| Variables | 7 | Clearly define all outcomes, exposures, predictors, potential confounders, and effect modifiers. Give diagnostic criteria, if applicable | 4-6 |  |
| Data sources/ measurement | 8* | For each variable of interest, give sources of data and details of methods of assessment (measurement). Describe comparability of assessment methods if there is more than one group | *4-6* |  |
| Bias | 9 | Describe any efforts to address potential sources of bias | 4-6 |  |
| Study size | 10 | Explain how the study size was arrived at | 4-6 |  |

Continued on next page

| Quantitative variables | 11 | Explain how quantitative variables were handled in the analyses. If applicable, describe which groupings were chosen and why | 6 |  |
| --- | --- | --- | --- | --- |
| Statistical methods | 12 | (*a*) Describe all statistical methods, including those used to control for confounding | 6 |  |
|  |  | (*b*) Describe any methods used to examine subgroups and interactions | 6 |  |
|  |  | (*c*) Explain how missing data were addressed | 6 |  |
|  |  | (*d*) *Cohort study*—If applicable, explain how loss to follow-up was addressed  *Case-control study*—If applicable, explain how matching of cases and controls was addressed  *Cross-sectional study*—If applicable, describe analytical methods taking account of sampling strategy | 6 |  |
|  |  | (*e*) Describe any sensitivity analyses | 6 |  |
| Results | | | | |
| Participants | 13* | (a) Report numbers of individuals at each stage of study—eg numbers potentially eligible, examined for eligibility, confirmed eligible, included in the study, completing follow-up, and analysed | 6 |  |
|  |  | (b) Give reasons for non-participation at each stage | 6-9 |  |
|  |  | (c) Consider use of a flow diagram |  |  |
| Descriptive data | 14* | (a) Give characteristics of study participants (eg demographic, clinical, social) and information on exposures and potential confounders | 6-9 |  |
|  |  | (b) Indicate number of participants with missing data for each variable of interest | 6-9 |  |
|  |  | (c) *Cohort study*—Summarise follow-up time (eg, average and total amount) | 6-9 |  |
| Outcome data | 15* | *Cohort study*—Report numbers of outcome events or summary measures over time | *6-9* |  |
|  |  | *Case-control study—*Report numbers in each exposure category, or summary measures of exposure |  |  |
|  |  | *Cross-sectional study—*Report numbers of outcome events or summary measures |  |  |
| Main results | 16 | (*a*) Give unadjusted estimates and, if applicable, confounder-adjusted estimates and their precision (eg, 95% confidence interval). Make clear which confounders were adjusted for and why they were included | 9 |  |
|  |  | (*b*) Report category boundaries when continuous variables were categorized | 9 |  |
|  |  | (*c*) If relevant, consider translating estimates of relative risk into absolute risk for a meaningful time period | 9 |  |

Continued on next page

| Other analyses | 17 | Report other analyses done—eg analyses of subgroups and interactions, and sensitivity analyses | 9 |  |
| --- | --- | --- | --- | --- |
| Discussion | | | | |
| Key results | 18 | Summarise key results with reference to study objectives | 9-10 |  |
| Limitations | 19 | Discuss limitations of the study, taking into account sources of potential bias or imprecision. Discuss both direction and magnitude of any potential bias | 12 |  |
| Interpretation | 20 | Give a cautious overall interpretation of results considering objectives, limitations, multiplicity of analyses, results from similar studies, and other relevant evidence | 12-13 |  |
| Generalisability | 21 | Discuss the generalisability (external validity) of the study results | 12-13 |  |
| Other information | |  | | |
| Funding | 22 | Give the source of funding and the role of the funders for the present study and, if applicable, for the original study on which the present article is based | 1 |  |

*Give information separately for cases and controls in case-control studies and, if applicable, for exposed and unexposed groups in cohort and cross-sectional studies.

**Note:** An Explanation and Elaboration article discusses each checklist item and gives methodological background and published examples of transparent reporting. The STROBE checklist is best used in conjunction with this article (freely available on the Web sites of PLoS Medicine at http://www.plosmedicine.org/, Annals of Internal Medicine at http://www.annals.org/, and Epidemiology at http://www.epidem.com/). Information on the STROBE Initiative is available at www.strobe-statement.org.
